# Supplementary material for: A Word of Caution—Potential Limitations of Pulmonary Artery Pressure Monitoring in Detecting Congestion Caused by Right-Sided Heart Failure
Source: Biomedicines. 2025 Jun 14;13(6):1469. doi: 10.3390/biomedicines13061469 (PMC12190875; doi:10.3390/biomedicines13061469)
Supplement: Supplementary file 1 [file biomedicines-13-01469-s001.zip › Supplementary Table.pdf]

Supplementary Table S1: Type of death

Patient-ID	Type of death
**3**	unknown
**6**	non cardiac non-cardiovascular
**9**	sudden cardiac death
**17**	non-cardiac non-cardiovascular
**20**	pump failure
**21**	sudden cardiac death
**22**	pump failure
**23**	pump failure
**30**	pump failure
**34**	pump failure
**38**	pump failure
