# Supplementary figures and images for: A Word of Caution—Potential Limitations of Pulmonary Artery Pressure Monitoring in Detecting Congestion Caused by Right-Sided Heart Failure
Source: Biomedicines. 2025 Jun 14;13(6):1469. doi: 10.3390/biomedicines13061469 (PMC12190875; doi:10.3390/biomedicines13061469)

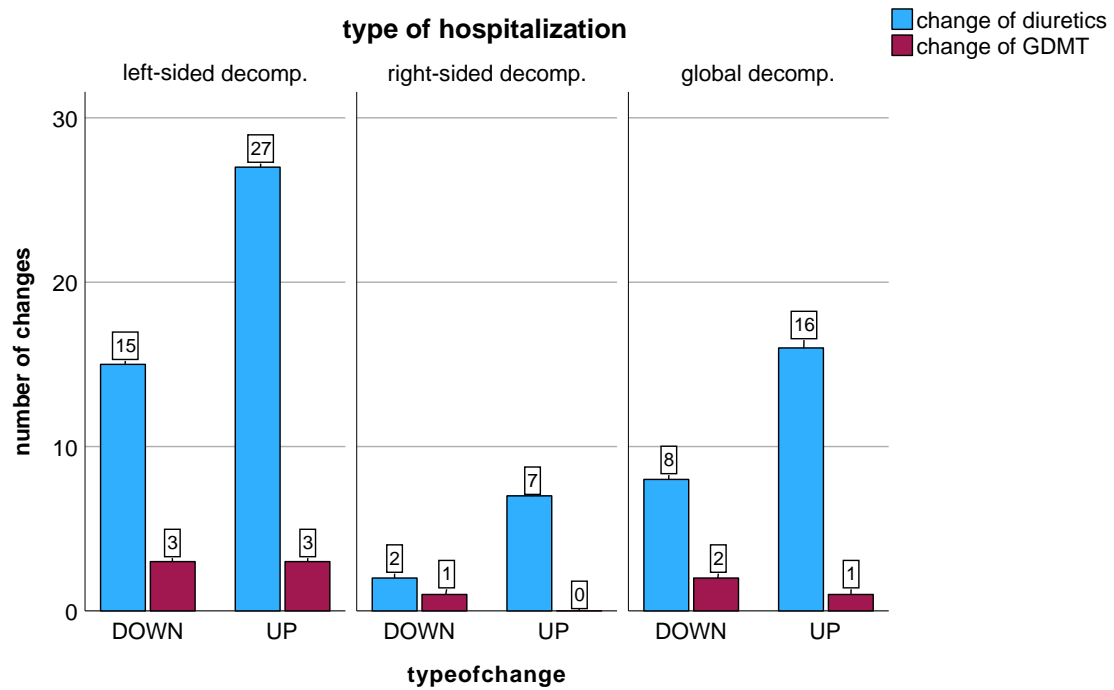

Supplement: Supplementary file 1 [file biomedicines-13-01469-s001.zip › Supplementary Figure S4.pdf]

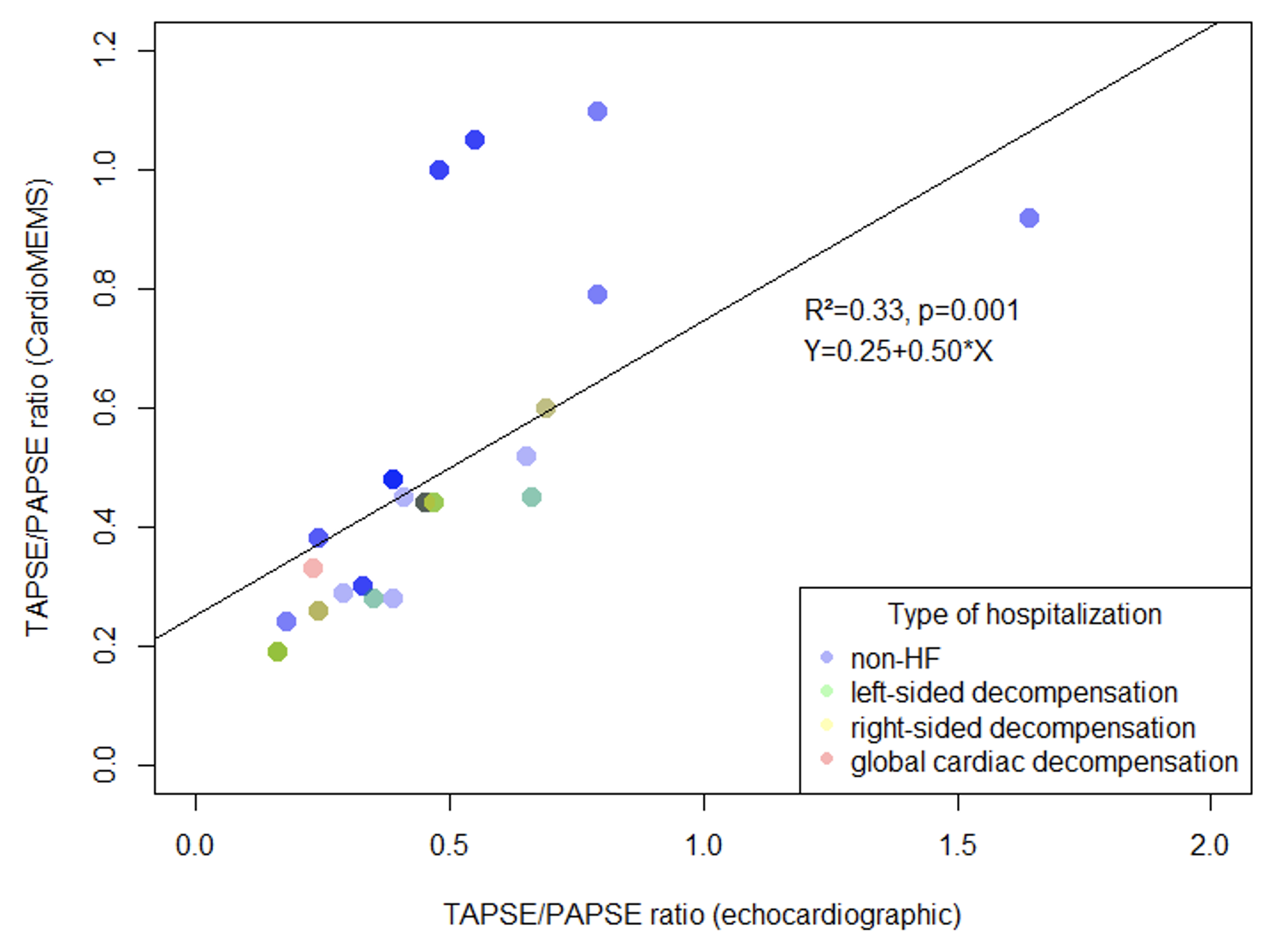

Supplement: Supplementary file 1 [file biomedicines-13-01469-s001.zip › Supplementary-Figure-S1.tiff]

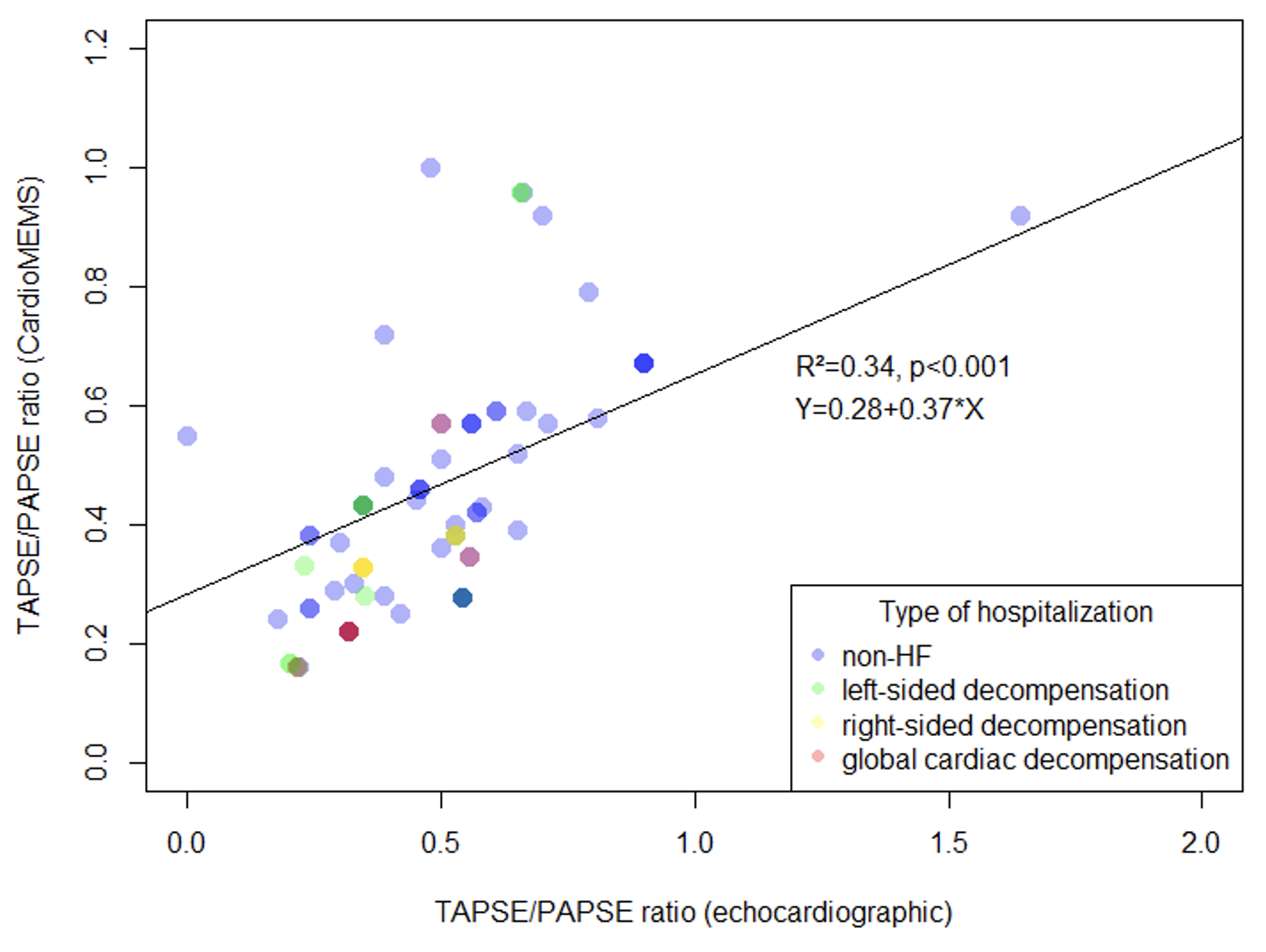

Supplement: Supplementary file 1 [file biomedicines-13-01469-s001.zip › Supplementary-Figure-S2.tiff]

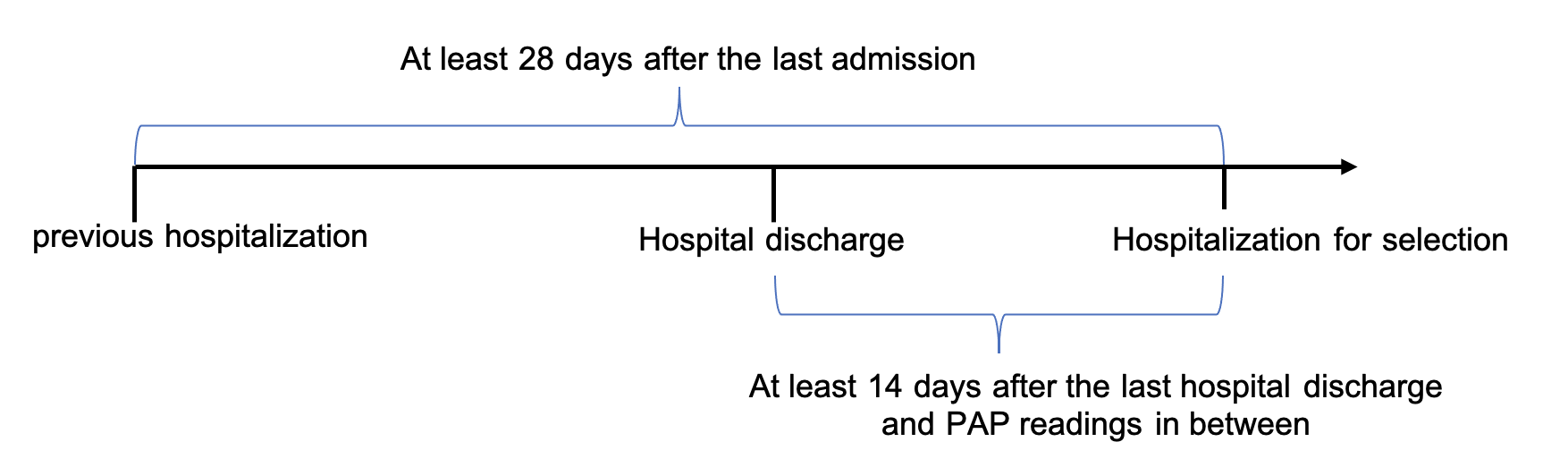

Supplement: Supplementary file 1 [file biomedicines-13-01469-s001.zip › Supplementary-Figure-S3.tiff]
